# A Stable Coordination Polymer Based on Rod-Like Silver(I) Nodes with Contiguous Ag-S Bonding

**DOI:** 10.3390/molecules25194548

**Published:** 2020-10-04

**Authors:** Harley D. Betts, Oliver M. Linder-Patton, Christopher J. Sumby

**Affiliations:** Centre of Advanced Nanomaterials and Department of Chemistry, University of Adelaide, Adelaide 5005, Australia; harley.betts@adelaide.edu.au (H.D.B.); oliver.linder-patton@adelaide.edu.au (O.M.L.-P.)

**Keywords:** coordination polymer, silver, thermal and chemical stability, silver chalcogenide

## Abstract

Silver(I)-based coordination polymers or metal-organic frameworks (MOFs) display useful antibacterial properties, whereby distinct materials with different bonding can afford control over the release of silver(I) ions. Such silver(I) materials are comprised of discrete secondary building units (SBUs), and typically formed with ligands possessing only soft or borderline donors. We postulated that a linker with four potential donor groups, comprising carboxylate and soft thioether donors, 2,5-bis (allylsulfanyl) benzene dicarboxylic acid (ASBDC), could be used to form stable, highly connected coordination polymers with silver(I). Here, we describe the synthesis of a new material, (Ag_2_(ASBDC)), which possesses a rod-like metal node-based 3D honeycomb structure, strongly π-stacked linkers, and steric bulk to protect the node. Due to the rod-like metal node and the blocking afforded by the ordered allyl groups, the material displays notable thermal and moisture stability. An interesting structural feature of (Ag_2_(ASBDC)) is contiguous Ag–S bonding, essentially a helical silver chalcogenide wire, which extends through the structure. These interesting structural features, coupled with the relative ease by which MOFs made with linear dicarboxylate linkers can be reticulated, suggests this may be a structure type worthy of further investigation.

## 1. Introduction

Metal-organic frameworks (MOFs) are crystalline, hybrid materials comprised of metal cluster nodes linked in a directional manner by organic linkers [1]. Metal ions or clusters from all blocks of the periodic table can be used as the metal nodes, while the organic linkers are often multidentate carboxylic acid or nitrogen-containing aromatic molecules [2]. Over the last twenty years, the MOF field has expanded greatly and many possible applications have been considered, including: storage/separation of gas and hydrocarbons [3,4], energy storage [5], sensing [6], catalysis [7], and drug delivery [8]. Regarding biomedical applications of MOFs, a wide range of uses have been investigated, including release/sensing of biologically-relevant gases (nitric oxide, carbon monoxide, hydrogen sulfide) [9,10,11]; encapsulation and release of drugs like ibuprofen, doxorubicin, caffeine, vancomycin, and silver nanoparticles [12,13]; and the incorporation of bioactive components into the MOF structure, such as silver, copper, or zinc ions at the metal cluster [14,15,16]. Biomedical applications of MOF-based composites are now attracting considerable attention [17].

**Figure 1:**
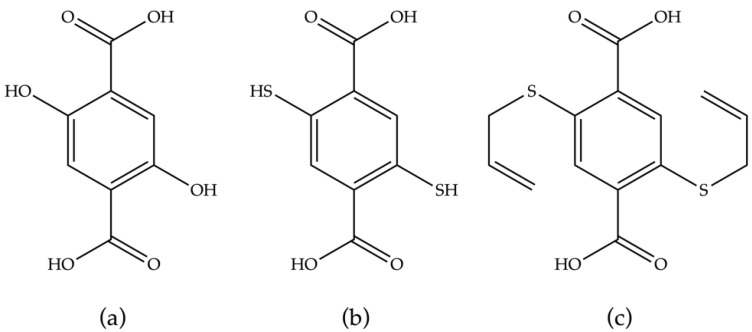
Linkers with high densities of donors: (**a**) 2,5-dihydroxybenzene dicarboxylic acid, (DOBDC); (**b**) 2,5-dimercaptobenzene dicarboxylic acid, (DMBDC); (**c**) 2,5-bis(allylsulfanyl)benzene dicarboxylic acid, (ASBDC).

The structures and properties of silver(I)-based MOFs and coordination polymers have been widely investigated, largely driven by the accommodating coordination preferences of silver ions and an interest in their antimicrobial properties [18,19]. Coordination polymers comprised of silver ions were used in the initial stages of the field to demonstrate a net-based design strategy [20], and the malleable coordination geometry of silver(I) has led to the formation of materials displaying pronounced structural flexibility [21,22]. Despite this interest, silver(I)-based coordination polymers are typically not inherently stable due to the relatively low bond energies. By tuning the ligand donors more stable materials can be encountered [23,24,25,26], but the stability of silver(I)-based MOFs is often conferred by relatively dense structures. General strategies that have been used to enhance the stability of MOFs include use of hydrophobic groups to disfavor hydrolysis [27], employing a double-wall structure to line the pores [28], and the use of rod-like secondary building units (SBUs) to impart structural rigidity in one direction [29]. Formation of rod-like SBUs is often achieved by employing linkers that possess a high density of donor groups [30], such as 2,5-dihydroxybenzene dicarboxylic acid (DOBDC, Figure 1) in the formation of the family of materials alternatively referred to as MOF-74-M, CPO-27, and M_2_(DOBDC) derivatives.

Herein, we report the synthesis of a new silver(I)-based coordination polymer (Ag_2_(ASBDC)), which possesses a rod-like metal node from a linker comprising carboxylate and thioether donors, 2,5-bis(allylsulfanyl)benzene dicarboxylic acid (ASBDC, Figure 1). Due to the rod-like metal node and the blocking afforded by the ordered allyl groups, the MOF displays notable thermal and moisture stability. The rod-like SBU present in (Ag_2_(ASBDC)) also provides contiguous Ag–S bonding, akin to a helical silver chalcogenide wire, which extends through the structure. Given these interesting structural features, and the relative ease by which MOFs made with linear aryl dicarboxylate linkers can be reticulated, we posit this may be a MOF topology worthy of further investigation.

## 2. Experimental Materials

All chemicals were purchased from Sigma Aldrich and used as received; silver trifluoroacetate, CAS: 2966-50-9, 98% purity; diethyl 2,5-dihydroxyl terephthalate, CAS: 5870-38-2, 97% purity; dimethyl thiocarbamoyl chloride, CAS: 16420-3-6, 97% purity; allyl bromide, CAS: 106-95-6, 99% purity. Where necessary, additional preparation of reagents, including drying of solvents was carried out by literature procedures.

### 2.1. Instrumentation and Methods

NMR spectra were recorded on a Varian 500 MHz spectrometer at 23 °C using a 5 mm probe. ^1^H NMR spectra were referenced to either TMS (0 ppm) or *d*_6_-DMSO (2.50 ppm). (Ag_2_(ASBDC)) samples were digested in DCl/*d*_6_-DMSO (2 drops/700 μL) at room temperature before NMR analysis. FTIR spectra were recorded on a Perkin Elmer Spectrum 100 spectrophotometer with ZnSe crystal windows. Powder X-ray diffraction data were collected using a Cu Kα (1.542 Å) source on a Bruker D8 Advanced X-ray powder diffractometer (parallel X-ray, capillary loaded). Samples run on the Bruker D8 were mounted in 0.5 mm diameter glass capillaries. Data were collected between 2θ of 2 to 52.94, phi rotation was 20 rotations per minute and 1 s exposure per step for 5001 steps. Raw data were converted to xye format, and WinPlotr 2006 software used for background subtraction. Simulated X-ray diffraction patterns were generated using Mercury from single crystal X-ray diffraction data. Scanning electron microscopy (SEM) images were collected on a Quanta 450 scanning electron microscope in secondary electron mode (spot size 4, 15 KeV). Energy dispersive X-ray analysis was collected with an Oxford Instruments Ultim Max 170 EDX attachment on the Quanta 450 (spot size 4, 15 KeV). Samples for SEM analysis were dry loaded onto adhesive carbon tabs on aluminium stubs and carbon coated (5 nm) prior to analysis.

### 2.2. Linker Synthesis

According to a literature procedure, 2,5-dimercaptobenzene dicarboxylic acid (DMBDC) was synthesized from diethyl 2,5-dihydroxybenzene dicarboxylate and dimethyl thiocarbamoyl chloride via a Newman Kwart rearrangement [31]. Thioetherification of DMBDC with allyl bromide produced 2,5-bis(allylsulfanyl)benzene dicarboxylic acid (ASBDC), which has previously been reported [32].

### 2.3. [Ag_2_(ASBDC)] Synthesis

Slow evaporation–single crystals. ASBDC (30 mg, 0.10 mmol) and silver trifluoroacetate (64.1 mg, 0.29 mmol) were separately dissolved in MeOH (2 × 2 mL). The two solutions were combined, resulting in a beige precipitate which was resolubilized by addition of ammonia (3% in MeOH, 4–5 drops), and the solution allowed to slowly evaporate in the dark for 2–3 days. The resultant colorless needles were washed with MeOH (3 × 2 mL) then acetone (3 × 2 mL), dried overnight under vacuum at 50 °C to give (Ag_2_(ASBDC)) as a white crystalline solid (16.2 mg, 31% based on ASBDC). Single crystals suitable for X-ray diffraction were removed and analysed prior to washing.

Microcrystalline powder. ASBDC (10 mg, 0.03 mmol) and silver trifluoroacetate (21.3 mg, 0.10 mmol) were separately dissolved in DMF (2 × 2 mL). The solution of ligand was added, dropwise, to the stirred solution of the silver salt and the mixture allowed to stir for a further 15 min after addition. The resultant beige suspension was isolated by centrifugation (10,000× *g*, 5 min), washed with DMF (3 × 1 mL) and acetone (3 × 1 mL), and dried under vacuum at 50 °C to give (Ag_2_(ASBDC)) as a beige microcrystalline powder (3.7 mg, 24% based on ASBDC). IR (neat): 3222, 1661, 1561, 1354, 1307, 1197, 1083, 920, 812, 726 cm^−1^.

## 3. Results and Discussion

**Figure 2:**
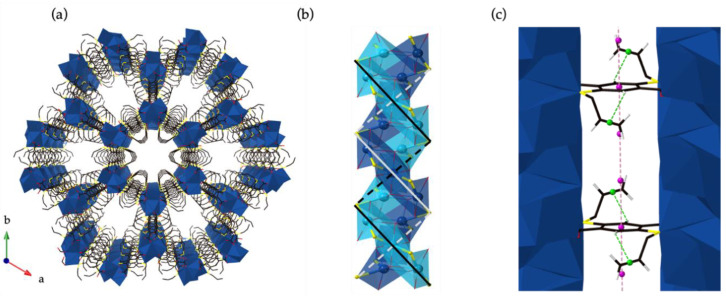
(**a**) The 3D structure of (Ag_2_(ASBDC)) showing the hexagonal pores that run along the *c*-axis of the framework with ~9 Å pore diameter. (**b**) (Ag_2_(ASBDC)) has rod-like nodes of silver ions along the *c*-axis of the structure with two contiguous, helical chains of (-Ag-S-)_n_ bonds, and (**c**) π-stacking involving the aryl rings (pink centroid-centroid separation 4.036 Å), and edge-to-face π-interactions of the allyl groups (green C-H···alkene centroid 2.677(5) Å) of the linker further contributing to the stability; only every third linker is visible for clarity, the pink centroids denote where the aryl ring of the linkers should be located.

The use of hard or borderline Lewis acids, such as zirconium(IV) or zinc(II), with linkers possessing thioether groups *ortho* to the dicarboxylate donors typically leads to the formation of UiO [32] or IRMOF structure types [33], in which the sulfur donors are not employed due to the coordination mismatch. For the mercapto-containing linker 2,5-dimercaptobenzene dicarboxylic acid (DMBDC), MOF-74, and UiO analogues were prepared with Fe and Zr as the metals, respectively [34,35]. In the case of the 2,5-bis(allylsulfanyl)benzene dicarboxylic acid (ASBDC) linker, we reasoned that the use of silver(I) as a metal node, with its coordination preference for soft thioether donors, might lead to more highly coordinated silver centers and a stable structure. The reaction of ASBDC with silver trifluoroacetate (AgTFA) in an ca. 3% ammonia in methanol solution, and evaporation of the solvent over 2–3 days, provided very small colorless hexagonal rod-shaped crystals (ca. 0.10 × 0.01 × 0.01 mm, see Figures S8 and S10) of (Ag_2_(ASBDC)) suitable for single crystal X-ray crystallography (parameters shown in Table S1). The same material could be formed instantly as an off-white microcrystalline powder by combination of solutions of ASBDC and AgTFA in dimethylformamide, where residual base facilitates rapid precipitation. The small quantity of ammonia added to the reaction used to form single crystals was needed to avoid instantaneous formation of the microcrystalline precipitate of (Ag_2_(ASBDC)]); ammonia is commonly used as a base in the synthesis of silver coordination polymers [16] as it acts as a base to deprotonate the ligand but also complexes the silver(I) ions, thereby modulating reactivity and facilitating crystal growth. Despite using ammonia in the synthesis, none of the reagent was detected in the (Ag_2_(ASBDC)) product. Attempts to synthesize analogous (Ag_2_(ASBDC)) materials using DMBDC or 2,5-bis(propylsulfanyl)benzene dicarboxylic acid as microcrystalline powders or single crystals, resulted in amorphous precipitates (data not shown). This potentially highlights the necessity of the allyl functionality of the linker. The FTIR spectrum of (Ag_2_(ASBDC)) (Figure S5) revealed slight red-shifting and broadening of observed stretches (relative to the free ASBDC linker); consistent with the high degree of inter- and intra-molecular, non-covalent bonding present in the material (vide infra) [36]. Acidic digestion of the material and analysis by ^1^H NMR spectroscopy confirmed the allyl groups of the ASBDC linker were unreacted after formation of (Ag_2_(ASBDC)) (Figure S6). Scanning electron microscopy and energy dispersive X-ray spectroscopy (EDX) analysis confirmed uniformity in colocalisation of the relevant elements (Ag, S, C, O) in a crystalline sample of (Ag_2_(ASBDC)) (Figures S8 and S9 and Table S3). The EDX Ag:S ratio also corroborated the Ag to linker ratio of 2:1, recorded in the crystallographic analysis. The crystal morphology was found to be hexagonal, reflecting the unit cell of the crystal structure (Figure S10).

(Ag_2_(ASBDC)) has a three-dimensional honeycomb structure possessing hexagonal pores (*R*-3*c* Figure 2a). The structure is comprised of silver ions forming three-fold symmetric one-dimensional helical chains, connected by ASBDC linkers (Figure 2b); the asymmetric unit comprises two silver ions chelated by sulfur and oxygen atoms from half an ASBDC linker (Figure S2). Pillars of silver atoms coordinated by carboxylate oxygen, and sulfur atoms from the ASBDC linkers, project along the *c*-axis. Two crystallographically independent silver S_2_O_4_ coordination spheres are present in the coordination polymer, both with distorted octahedral geometries, however, they differ in positioning of the sulfur donors (Figure 2b); the S-Ag-S bond angles are either near linear (S-Ag-S angle: 169.1(7)°) or at right angles (S-Ag-S angle: 88.4(6)°) for the exterior and centrally positioned silver ions in the rod-like SBUs. These coordination spheres give rise to contiguous Ag–S bonding that intertwines around the rod-like SBU. Similar, non-discrete helical (–Ag–S–)_n_ chains have been observed for MOFs formed from silver(I), fluorinated BDC derivatives, and tert-butylthiolate [27]; but this represents a rare example where these chains form in a two component material. The exposed and more distorted 6-coordinate silver atoms on the periphery of the node have longer Ag–O bonds, and are further protected by the allyl groups, which protrude from the linker adjacent to the silver centers.

The rod-like nodes in (Ag_2_(ASBDC)) are coordinated to three carboxylate linkers at each repeat, giving rise to hexagonal pores with pore diameters of ~9 Å, measured from the carbon atoms of the allyl thioether groups. The structure of (Ag_2_(ASBDC)) bears significant similarities to those of M-MOF-74 (M = Zn, Ni, Co, Mg, Mn, Fe); a family of stable materials formed from 2,5-dihydroxybenzene dicarboxylate (DOBDC) [37,38]. In MOF-74, one-dimensional, infinite, helical M_2_O_2_(COO)_2_ SBUs are bridged by the DOBDC linker to form a similar three-dimensional honeycomb structure, with infinite rod-like SBUs [39]. The pores of M-MOF-74 are considerably larger (ca. 14 Å), as the ligands are aligned closer to the direction of the channels and lack the allyl groups present in (Ag_2_(ASBDC)). Due to the divalent nature of the metals in MOF-74 that material only has a single metal atom position in the asymmetric unit, which has a square pyramidal geometry coordinated by five oxygen atoms; conversely (Ag_2_(ASBDC)) has two Ag(I) sites that act to enlarge the diameter of the node.

The crystal structure of (Ag_2_(ASBDC)) reveals the material is likely stabilized by π-stacking interactions. The benzene rings of the ASBDC linkers are separated by 4.036 Å (Figure 2c), within the distance range ascribed to π-stacking interactions [40]. When viewed down the *c*-axis, the hexagonal pores are lined by interdigitated allyl groups from the ASBDC linkers. The pendant allylsulfanyl ether units appear to form intermolecular π–π contacts with nearby ASBDC thioether groups (Figure 2c). Similar interdigitation of the pendant allyl functionality of ASBDC was present along both the *a*- and *b*-axes in the molecular crystal structure (Figure S4a,b). Furthermore, close contacts between the pendant allyl group and the linker benzene core of (Ag_2_(ASBDC)), forming edge-to-face π-interactions with distances of 2.678 Å (Figure 2c).

**Figure 3:**
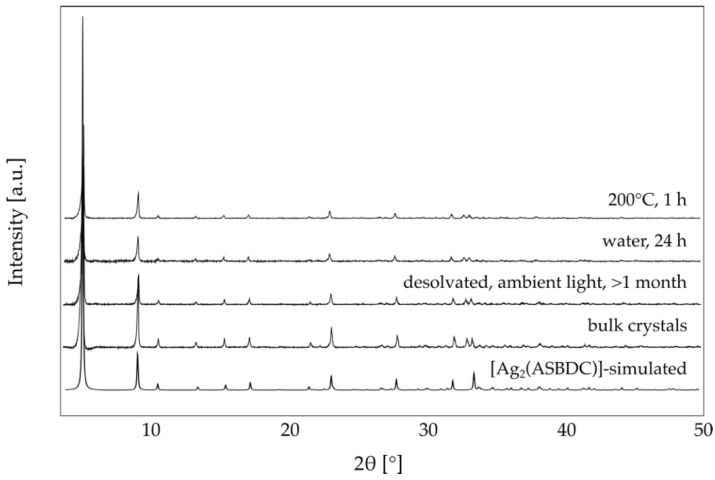
Powder X-ray diffraction data (Cu Kα, λ = 1.5418 Å) for (Ag_2_(ASBDC)); simulated patterned from crystal structure; bulk crystalline material generated by slow MeOH evaporation; desolvated for >1 month in air and ambient light; soaked in deionized water for 24 h; heated at 200 °C in air for 1 h. See Figure S1 for additional PXRD data demonstrating the stability of (Ag_2_(ASBDC)).

To confirm phase purity of (Ag_2_(ASBDC)), powder X-ray diffraction (PXRD) data were collected for a sample of single crystals, and the microcrystalline powder obtained by the room temperature synthesis (Figure 3). While not expected to be as porous as related M-MOF-74 structures, (Ag_2_(ASBDC)) still possesses potential solvent accessible void volume, which was calculated to be 959 Å^3^ (ca. 13% of the unit cell volume). This observation prompted further consideration of the stability of (Ag_2_(ASBDC)). The material retained crystallinity when an acetone exchanged sample was heated at 50, 100, 150, and 200 °C for 1 h under standard atmosphere and pressure (Figure 3 and Figure S1). Moreover, due to the hydrophobic nature of the pores, (Ag_2_(ASBDC)) retained crystallinity after soaking in water for 24 h. In addition, the material retained crystallinity when desolvated in air, and after exposure to ambient light for >1 month (Figure 3 and Figure S1).

Materials Studio was used to calculate a surface area for (Ag_2_(ASBDC)) of ca. 149 m^2^/g, based on the single crystal density (void fraction ca. 13%; pore volume ca. 0.065 cm^3^/g using a 1.84 Å radii probe, Figure S11, Table S4). This prompted us to examine activation conditions and to attempt to obtain an experimental surface area for the material. However, despite multiple attempts to activate (Ag_2_(ASBDC)) using a range of activation solvents (acetone, and CH_2_Cl_2_ and *n*-pentane) and conditions (typically 50 °C to 100 °C under high vacuum), 77 K N_2_ or 195K/293K CO_2_ isotherms showed the material had no measurable porosity (data not shown). To ascertain that the (Ag_2_(ASBDC)) structure was retained after activation, pre- and post-experiment PXRD data were collected showing that the crystallinity of the material remained largely unchanged (Figure S1). Given the lack of adsorption, the success of the activation procedure was also checked by examining the pore contents in air-dried and “activated” samples of (Ag_2_(ASBDC)). The activated material was digested in DCl/*d*_6_-DMSO, and the ^1^H NMR spectra recorded showed a small, but considerably reduced amount, of *n*-pentane trapped in the sample (heated at 100 °C under vacuum overnight) compared to the air dried sample (Figure S7). Thus, the lack of measurable porosity is more likely attributable to the narrow pore apertures (ca. 9 Å) and/or disordering of the allyl groups upon activation, blocking the pores.

## 4. Conclusions

This contribution describes the synthesis of a rod-based coordination polymer with a honeycomb 3D structure formed from Ag(I) ions and a thioether substituted dicarboxylate linker. The helical rod-like SBU of the structure, coupled with the strongly π-stacked linkers and the hydrophobic allyl groups lining the pores, accord the material significant stability, despite the typically weak Ag–O bonding. Unfortunately, the allyl groups also appear to preclude the material from showing permanent porosity. The topology that is encountered displays a number of interesting structural features, including silver chalcogenide (–Ag–S–)_n_ chains, which extend through the structure. Moreover, given the ease by which MOFs based on linear dicarboxylate linkers can be reticulated, coupled with the understanding that the thioether substituent plays an important stabilizing (and even templating) role, we propose that there is scope to form a family of materials based on the structure reported here. Isoreticulation of (Ag_2_(ASBDC)), resulting in a larger diameter pore, could allow the realization of permanent porosity, and provide access to the thioester and allyl functionalities, likely useful for sequestration of heavy metals [32,33].

## Supplementary Materials

The following are available online, data on the synthesis of ASBDC, additional PXRD data, SCXRD data and tables, IR and NMR spectra for the MOF, SEM data.
