# Supplementary material for: A Stable Coordination Polymer Based on Rod-Like Silver(I) Nodes with Contiguous Ag-S Bonding
Source: Molecules. 2020 Oct 4;25(19):4548. doi: 10.3390/molecules25194548 (PMC7583003; doi:10.3390/molecules25194548)
Supplement: Supplementary file 1 [file molecules-25-04548-s001.pdf]

Supplementary Information for:

A stable coordination polymer based on rod-like silver(I) nodes with contiguous Ag-S bonding

Harley D. Betts[1], Oliver M. Linder-Patton[1] and Christopher J. Sumby[1,*]

[1] Centre of Advanced Nanomaterials and Department of Chemistry, University of Adelaide
[*] Correspondence: christopher.sumby@adelaide.edu.au

1. Selected Data for the Synthesis of 2,5-Bis(allylsulfanyl)benzene Dicarboxylic Acid (ASBDC)

Diethyl 2,5-bis(dimethylthiocarbamoyloxy)benzene dicarboxylate.[1] Fluffy white solid (3.41 g, 99%). ^{1}H NMR (500 MHz, $CDCl_3$): δ 7.73 (s, 2H), 4.30 (q, *J* = 7.1 Hz, 4H), 3.46 (s, 6H), 3.40 (s, 6H), 1.33 (t, *J* = 7.1 Hz, 6H); ^{13}C NMR (126 MHz, $CDCl_3$): δ187.13, 163.05, 150.55, 128.62, 127.82, 61.67, 42.44, 39.08, 14.20 ppm; consistent with literature.

Diethyl 2,5-bis(dimethylthiocarbamoylsulfanyl)benzene dicarboxylate.[1] Pale brown platy crystals (0.93 g, 92%). ^{1}H NMR (500 MHz, $CDCl_3$): δ 8.11 (s, 2H), 4.34 (q, *J* = 7.1 Hz, 4H), 3.17 (s, 6H), 3.02 (s, 6H), 1.35 (t, 6H); ^{13}C NMR (126 MHz, $CDCl_3$): δ165.40, 165.36, 138.80, 137.24, 137.24, 131.00, 61.73, 37.16, 14.25 ppm; consistent with literature.

2,5-Dimercaptobenzene dicarboxylic acid (DMBDC).[1] Yellow solid (1.46 g, 98%). ^{1}H NMR (500 MHz, DMSO-d^6): δ 8.03 (s, 2H); ^{13}C NMR (126 MHz, $CDCl_3$): δ166.87, 133.37, 130.13 ppm; consistent with literature.

Dimethyl 2,5-dimercaptobenzene dicarboxylate.[2] Pale-yellow solid (1.10 g, 99%). ^{1}H NMR (500 MHz, $CDCl_3$): δ 7.96 (s, 2H), 4.68 (s, 2H), 3.94 (s, 6H); consistent with literature.

Dimethyl 2,5-bis(allylsulfanyl)benzene dicarboxylate.[2] Pale yellow solid (0.064 g, 92%). ^{1}H NMR (500 MHz, $CDCl_3$): δ7.90 (s, 2H), 5.88 (ddt, *J* = 16.9, 10.1, 6.8 Hz, 2H), 5.32 (d, *J* = 17.0 Hz, 4H), 5.19 (d, *J* = 10.1 Hz, 4H), 3.94 (s, 6H), 3.62 ppm (d, *J* = 6.8 Hz, 4H); ^{13}C NMR (126 MHz, $CDCl_3$): δ166.26, 138.32, 130.48, 129.82, 127.60, 115.60, 52.65, 31.07 ppm; consistent with literature.

2,5-Bis(allylsulfanyl)benzene dicarboxylic acid (ASBDC).[2] Yellow solid (0.28 g, 78%). ^{1}H NMR (500 MHz, DMSO-d^6): δ 13.43 (s, 2H), 7.80 (s, 2H), 5.84 (ddt, *J* = 16.6, 10.1, 6.8 Hz, 2H), 5.29 (d, *J* = 17.0 Hz, 4H), 5.15 (d, *J* = 10.0 Hz, 4H), 3.65 (d, *J* = 6.2 Hz, 4H); ^{13}C NMR (126 MHz, DMSO-d^6): δ166.77, 136.79, 131.06, 126.79, ppm; consistent with literature. IR: 3406, 2924, 1709, 1429, 1296, 1082, 956, 878, 822, 775, 727, 609 cm^{-1}.

ASBDC single crystals

A solution of ASBDC (5 mg, 0.02 mmol) in MeOH (5 mL) was allowed to evaporate slowly for ~1 week, yielding single yellow platy crystals suitable for X-ray diffraction.

2. Powder X-ray Diffraction Data for [Ag_2(ASBDC)]

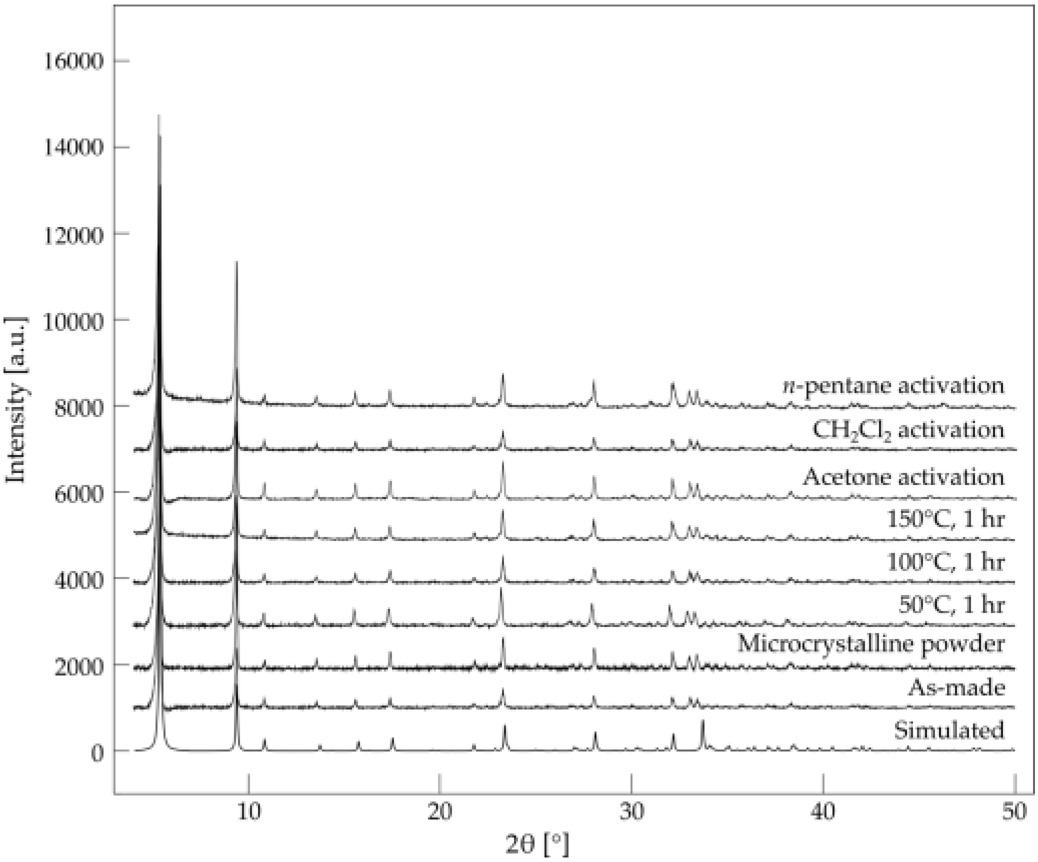

Figure S1: Powder X-ray diffraction data (Cu Kα, λ = 1.5418 Å) for [Ag_2(ASBDC)]; simulated patterned from crystal structure; as-made material generated by slow MeOH evaporation; microcrystalline powder formed at room temperature; heated at 50, 100, 150°C for 1 hour in air from acetone; attempted activation from n-pentane for 1 hr, 50°C.

3. Single crystal X-ray diffraction

Single crystals of **[Ag_2(ASBDC)]** were mounted under paratone-N oil on a MiTeGen crystal mount, and X-ray diffraction data was collected at 100 K on the MX2 beamline at the Australian Synchrotron using the Blue-ice software interface, λ=0.71073 Å.[3] The data set was corrected for absorption and the structure solved using SHELXT and refined by full matrix least-squares on F2 by SHELXL, interfaced through the programs X-Seed and Olex.[4-7] In general, all non-hydrogen atoms were refined anisotropically, and hydrogen atoms were included as invariants at geometrically estimated positions. Details of data collection and structure refinement are given in Table S1.

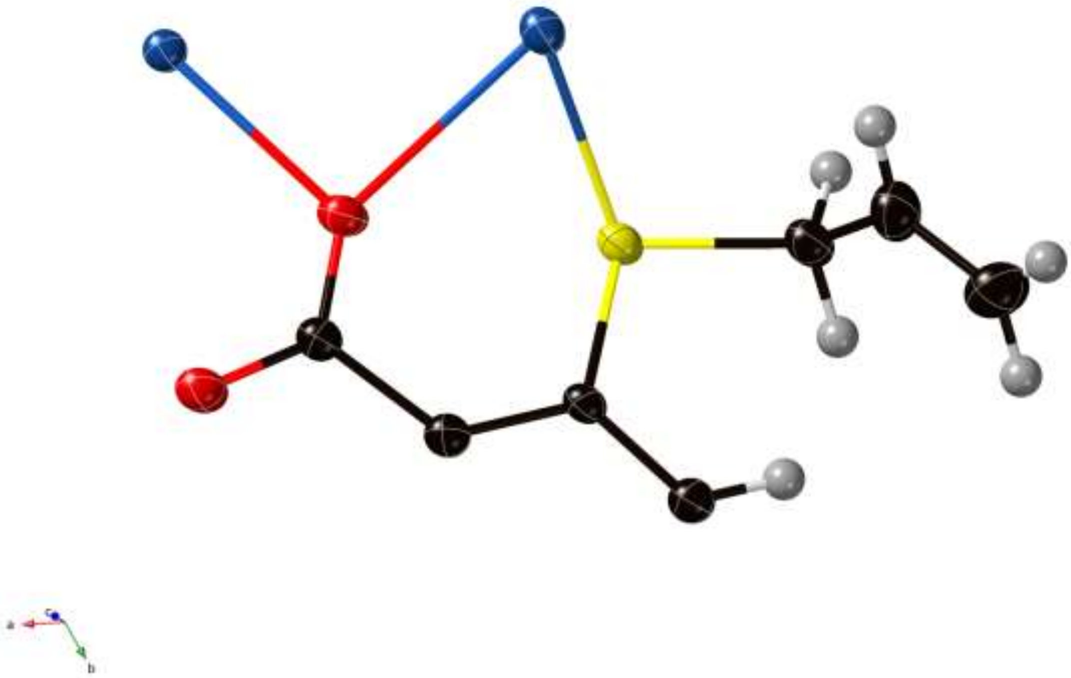

Figure S2: Asymmetric unit of [Ag_2(ASBDC)]. Thermal ellipsoids shown at the 50% probability level. Ag – navy, sulfur – yellow, oxygen – red, carbon – black and hydrogen – grey.

Table S1: Crystal data and structure refinement of [Ag_2(ASBDC)]

Identification code	[Ag_2(ASBDC)]
CCDC #	2032265
Empirical formula	$C_{14}H_{12}Ag_2O_4S_2$
Formula weight	524.10
Temperature/K	100(2)
Crystal system	trigonal
Space group	*R*-3*c*
a/Å	32.609(5)
b/Å	32.609(5)
c/Å	8.0720(16)
α/°	90
β/°	90
γ/°	120
Volume/Å^3	7434(3)
Z	18
ρ_{calc}g/cm^3	2.107
μ/mm^{-1}	2.635
F(000)	4572.0
Crystal size/mm^3	0.1 × 0.01 × 0.01
Radiation	Synchrotron (λ = 0.71073)
2Θ range for data collection/°	2.498 to 57.796
Index ranges	$-36 \leq h \leq 37$, $-43 \leq k \leq 43$, $-10 \leq l \leq 10$
Reflections collected	30658
Independent reflections	1997 [R_{int} = 0.1295, R_{sigma} = 0.0458]
Data/restraints/parameters	1997/0/101
Goodness-of-fit on F^2	1.047
Final R indexes [I>=2σ (I)]	R_1 = 0.0519, wR_2 = 0.1376
Final R indexes [all data]	R_1 = 0.0666, wR_2 = 0.1485
Largest diff. peak/hole / e Å^{-3}	1.49/-1.93

Single crystals of **ASBDC** were mounted under paratone-N oil on a MiTeGen crystal mount, and X-ray diffraction data was collected an Oxford Xcalibur diffractometer fitted with an Eos detector at 150(2) K. The data set were corrected for absorption, the structure solved by direct methods using SHELXS and refined by full matrix least-squares on F2 by SHELXL, interfaced through the programs X-Seed and Olex.[4-7] In general, all non-hydrogen atoms were refined anisotropically, and hydrogen atoms were included as invariants at geometrically estimated positions. Details of data collection and structure refinement are given in Table S2. The allyl group is disordered and a series of restraints were used to stabilize the refinement.

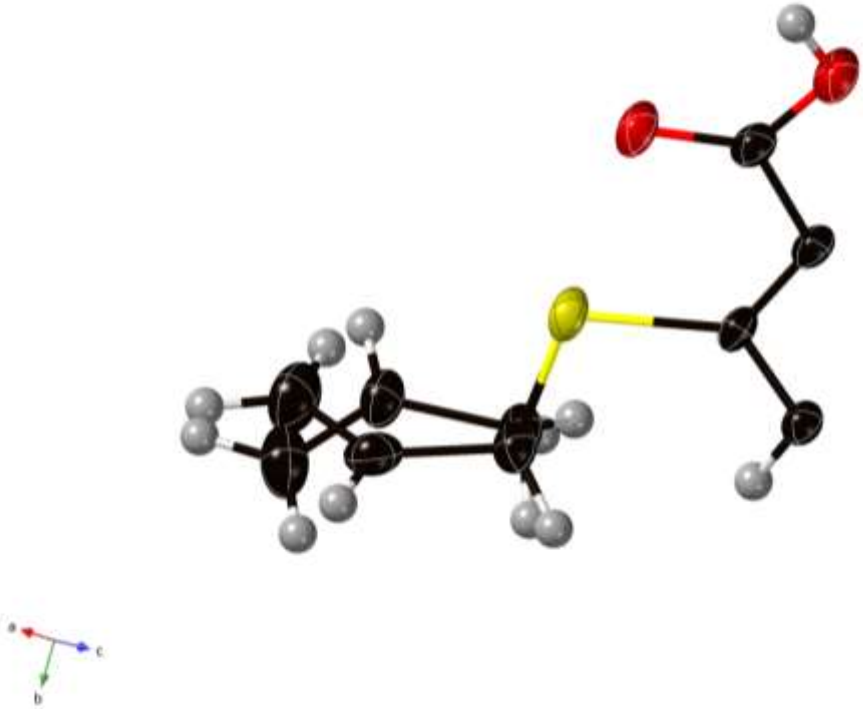

Figure S3: Asymmetric unit of ASBDC showing disorder in the pendant allyl group. Thermal ellipsoids shown at the 50% probability level. Sulfur – yellow, oxygen – red, carbon – black and hydrogen – grey.

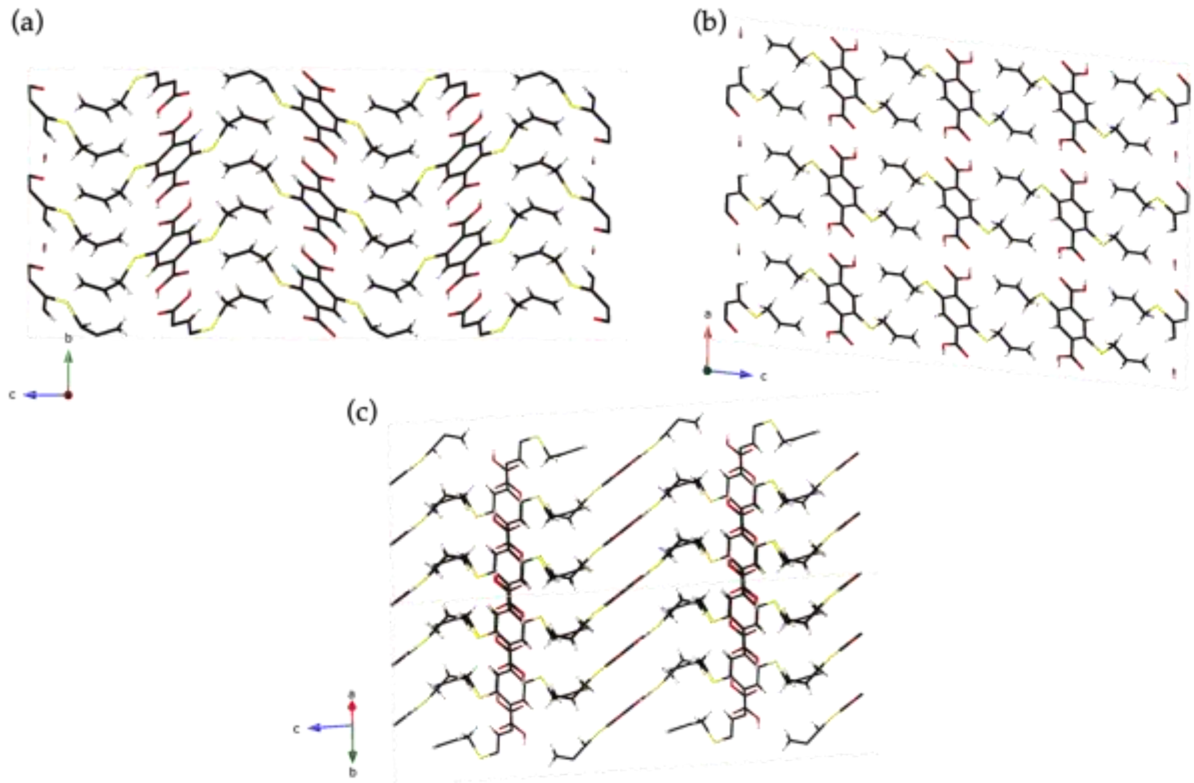

Figure S4: Crystal packing of ASBDC, showing interdigitation of pendant allyl groups in two axial directions (a) and (b); and intramolecular pi-pi interactions, and hydrogen bonding (c). Disorder was present in the allyl groups, however, for clarity only one phase was shown.

Table S2: Crystal data and structure refinement for ASBDC

Identification code	ASBDC
CCDC #	2032266
Empirical formula	$C_{14}H_{14}O_4S_2$
Formula weight	310.37
Temperature/K	150(2)
Crystal system	monoclinic
Space group	$P2_1/c$
a/Å	7.9692(4)
b/Å	5.2335(3)
c/Å	17.0625(12)
α/°	90
β/°	95.488(6)
γ/°	90
Volume/Å^3	708.36(7)
Z	2
ρ_{calc}g/cm^3	1.455
μ/mm^{-1}	0.385
F(000)	324.0
Crystal size/mm^3	0.35 × 0.07 × 0.02
Radiation	MoKα (λ = 0.71073)
2Θ range for data collection/°	7.358 to 58.588
Index ranges	-10 ≤ h ≤ 10, -7 ≤ k ≤ 6, -23 ≤ l ≤ 23
Reflections collected	6515
Independent reflections	1703 [R_{int} = 0.0605, R_{sigma} = 0.0754]
Data/restraints/parameters	1703/22/114
Goodness-of-fit on F^2	1.051
Final R indexes [I>=2σ (I)]	R_1 = 0.0478, wR_2 = 0.0935
Final R indexes [all data]	R_1 = 0.0866, wR_2 = 0.1080
Largest diff. peak/hole / e Å^{-3}	0.32/-0.28

4. FTIR Spectra of [Ag_2(ASBDC)] and ASBDC

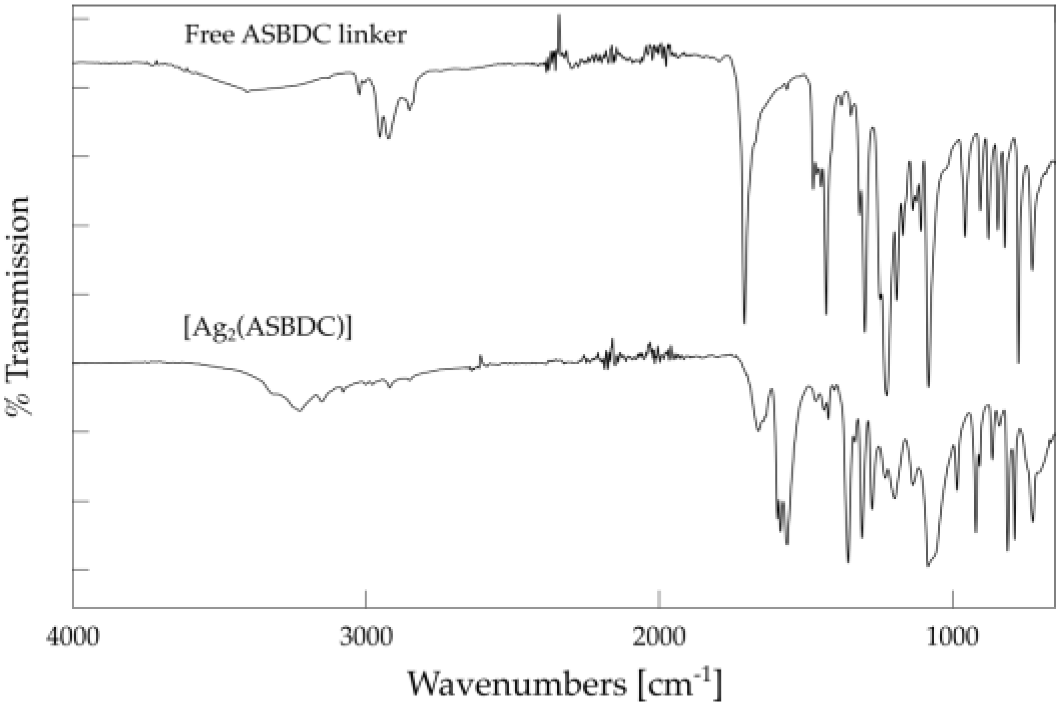

Figure S5: Fourier transform infrared spectra of neat free ASBDC linker and [Ag_2(ASBDC)].

5. ^{1}H NMR Spectrum of digested [Ag_2(ASBDC)]

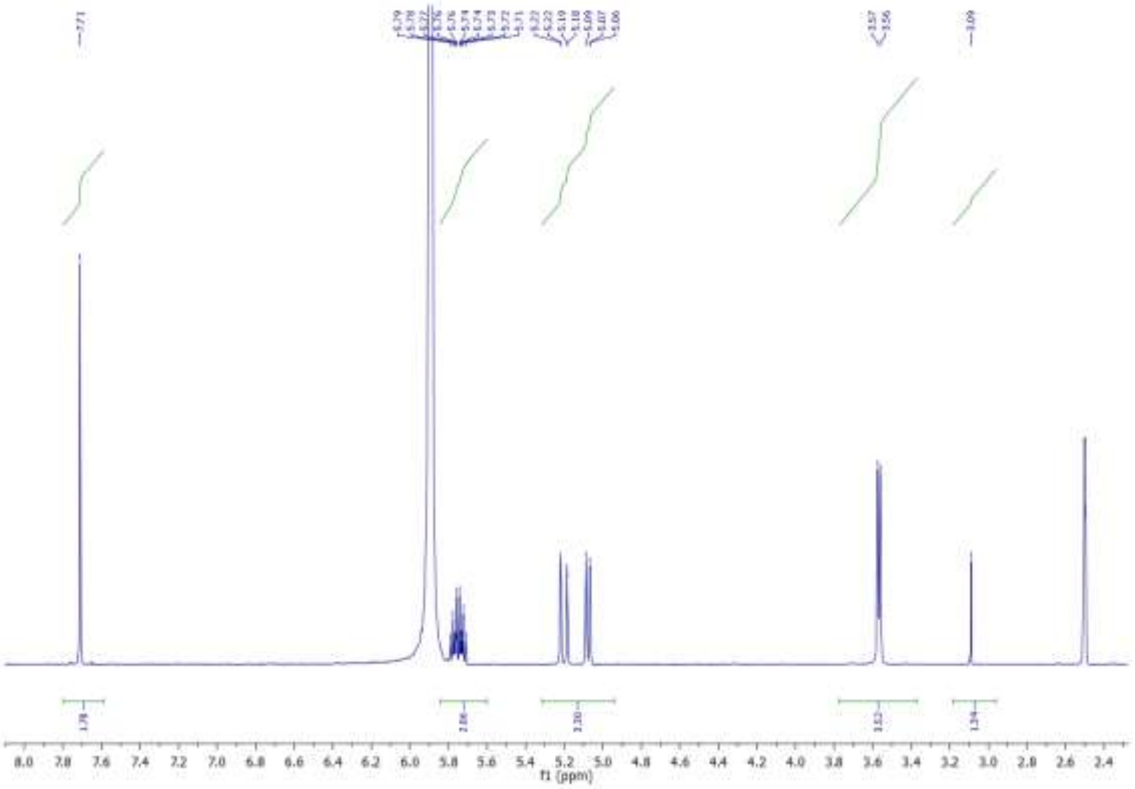

Figure S6: ^{1}H NMR spectrum of ASBDC linker, liberated by digestion of [Ag_2(ASBDC)] in DCl/d_6-DMSO. The presence of the complex multiplet at 5.75 ppm indicates the allyl functionality was unreacted during material synthesis.

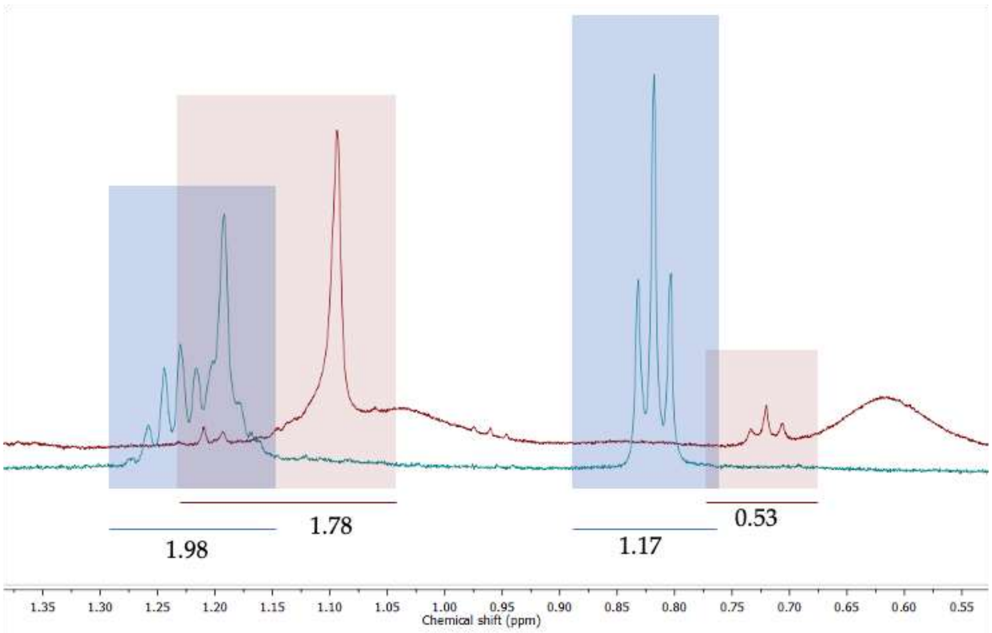

Figure S7: Stacked ^{1}H NMR spectra of ASBDC from digested [Ag_2(ASBDC)] soaked in *n*-pentane and air dried (blue) and 'activated': heated at 100°C, overnight, under vacuum (red). Showing characteristic ^{1}H NMR peaks and integrations for *n*-pentane between 0.7 and 1.3 ppm in d_6-DMSO).[8] Peak intensities were normalized between the spectra relative to the aryl H peak at ~7.7 ppm.

6. Scanning electron microscopy and energy dispersive X-ray spectroscopy analysis

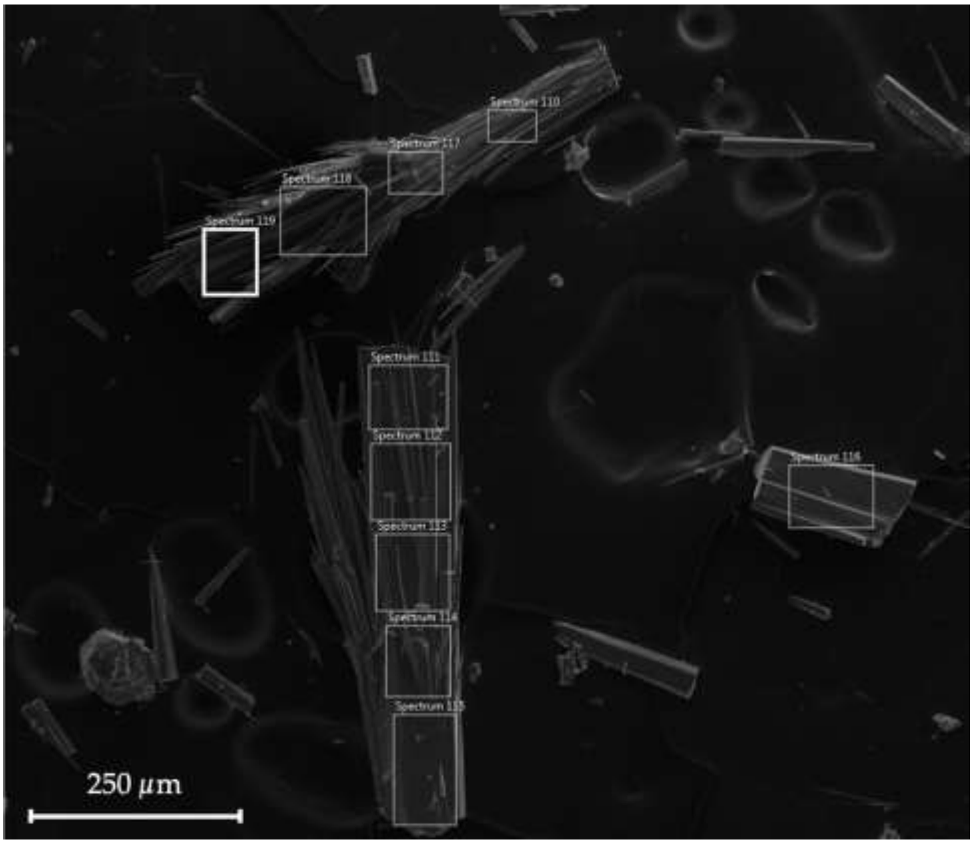

Figure S8: Scanning electron microscopy image of [Ag_2(ASBDC)] crystals (these have been crushed to form small clumps and isolated crystals).

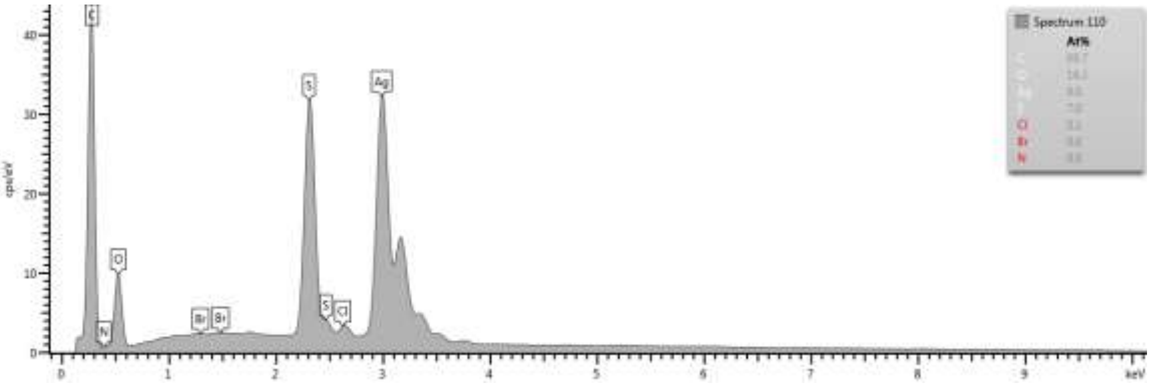

Figure S9: Energy dispersive X-ray spectroscopy spectrum taken from area 110 shown in Figure S8.

Table S3: Statistical analysis of EDX spectra collected from areas identified in Figure S8, n=10.

Element	Atomic %	Std dev	Std error
C	67.19	1.09	0.35
O	17.20	0.71	0.22
S	7.22	0.52	0.16
Ag	8.37	0.46	0.15

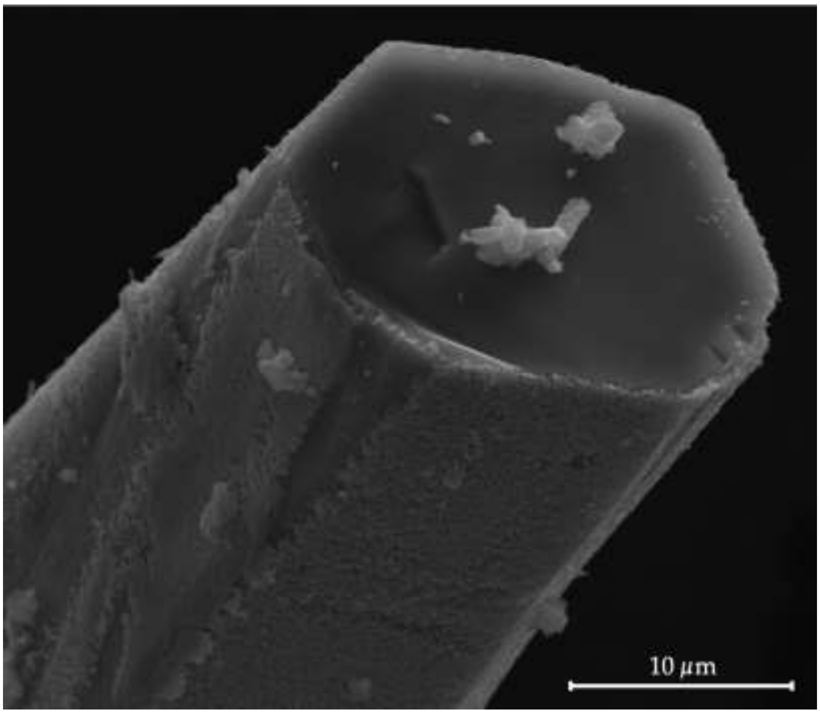

Figure S10: SEM image highlighting the hexagonal morphology of the [Ag_2(ASBDC)] crystals.

7. Materials Studio Calculations

The Materials Studio Software package was used to examine the porosity of [Ag_2(ASBDC)]. Representations of the accessible solvent surface area (using a 1.84 Å probe) and the Connolly surface area are provided in **Figure S11**.

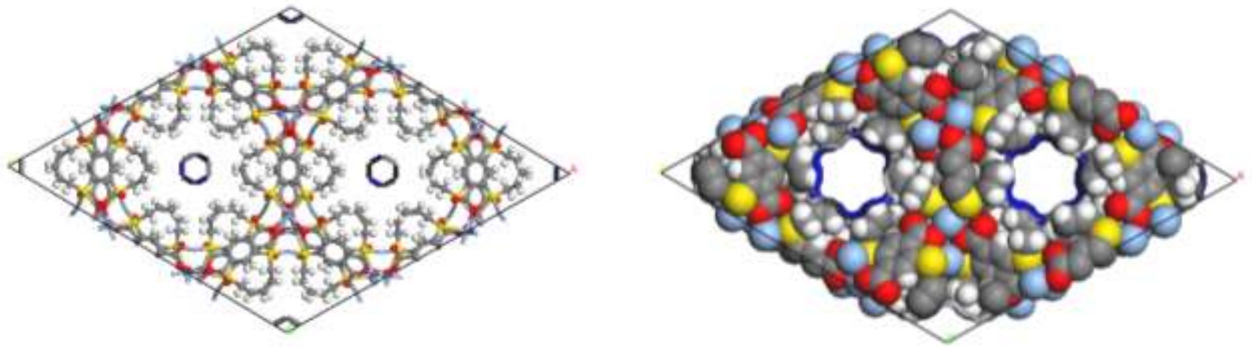

Figure S11: Representations of (a) the accessible solvent surface area (using a 1.84 Å probe) and (b) the Connolly surface [Ag_2(ASBDC)] are shown (perspective looking down the c-axis). For the surface area representations, blue is the free space side and grey is the framework face. Atom colours: pale blue – silver, yellow – sulfur, red – oxygen, carbon – grey and hydrogen – white. The unit cell is shown.

Table S4: Calculated parameters describing the structure of [Ag_2(ASBDC)]

Parameter	[Ag_2(ASBDC)]
Surface Area per cell (Å^2)	233.05
Density (g/cm^3)	2.11
Cell Volume (Å^3)	7433.39
Surface Area (calc) (m^2/g)	148.8
Cell Free Volume (Å^3)	1028.59
Void Fraction (%)	13.8
Pore Volume (cm^3/g)	0.066

8. References

1. Skowron, P.-T.; Dumartin, M.; Jeamet, E.; Perret, F.; Gourlaouen, C.; Baudouin, A.; Fenet, B.; Naubron, J.-V.; Fotiadu, F.; Vial, L.; Leclaire, J., On-demand cyclophanes: Substituent-directed self-assembling, folding, and binding. *J. Org. Chem.* **2016,** *81* (2), 654-661.
2. He, J.; Zha, M.; Cui, J.; Zeller, M.; Hunter, A. D.; Yiu, S. M.; Lee, S. T.; Xu, Z., Convenient detection of Pd(II) by a metal-organic framework with sulfur and olefin functions. *J. Am. Chem. Soc.* **2013,** *135* (21), 7807-10.
3. McPhillips, T. M.; McPhillips, S. E.; Chiu, H.-J.; Cohen, A. E.; Deacon, A. M.; Ellis, P. J.; Garman, E.; Gonzalez, A.; Sauter, N. K.; Phizackerly, R. P.; Soltis, S. M.; Kuhn, P., Blu-Ice the distributed control system: Software for data acquisition and instrument control at macromolecular crystallography beamlines. *J. Synchrotron Rad.* **2002,** *9*, 401-406.
4. Sheldrick, G. M., Phase annealing in SHELX-90: Direct methods for larger structures. *Acta Cryst.* **1990,** *A46*, 467-473.
5. Sheldrick, G. M., SHELXL-2014. University of Göttingen: Göttingen, Germany, 2014.
6. Barbour, L. J., X-Seed - A software for supramolecular crystallography. *J. Supramol. Chem.* **2003,** *1* (4-6), 189-191.
7. Dolomanov, O. V.; Bourhis, L. J.; Gildea, R. J.; Howard, J. A. K.; Puschmann, H., OLEX2: a complete structure solution, refinement and analysis program. *J. Appl. Cryst.* **2009,** *42* (2), 339-341.
8. Fulmer, G. R.; Miller, A. J. M.; Sherden, N. H.; Gottlieb, H. E.; Nudelman, A.; Stoltz, B. M.; Bercaw, J. E.; Goldberg, K. I., NMR Chemical Shifts of Trace Impurities: Common Laboratory Solvents, Organics, and Gases in Deuterated Solvents Relevant to the Organometallic Chemist. *Organometallics* **2010,** *29* (9), 2176-2179.
